# PROFESSIONALIZING GERONTOLOGY AND CREDENTIALING GERONTOLOGISTS

**DOI:** 10.1093/geroni/igae098.0818

**Published:** 2024-12-31

**Authors:** Donna Schafer

**Affiliations:** National Association for Professional Gerontologists, Healdsburg, California, United States

## Abstract

There are many reasons people want to work with older adults. However, there is no guarantee that these motivated people will enroll in a gerontology degree program. Many do not know about Gerontology; others are not confident they can find a job with a gerontology degree. Those who do graduate from gerontology degree programs may be frustrated in competing with graduates of other programs who are able to obtain licenses or with “graduates” of weekend workshops producing “gerontologists.” It is ironic that at a time when we need more people to work with older adults, gerontology degree programs often struggle to enroll students. Low enrollments threaten the survival of existing programs. Institutional perceptions about uncertain enrollments inhibit the development of new degree programs. We have not done a good job “marketing” our field either inside or outside academia. The National Association for Professional Gerontologists (NAPG) was founded in 2005 to address some of these problems. NAPG credentials the educational and practice experience of gerontology professionals using the AGHE Standards and Guidelines and the Gerontology Competencies for Undergraduate and Graduate Education. Thus far, NAPG has credentialed over 350 practicing gerontology professionals. We need to do more. NAPG proposes an effort to develop partnerships among like-minded organizations to promote the field and to attract funding to re-energize it. A coalition of partners needs to work with the employer community to convey the value of a gerontology education and ensure that graduates have the skills employers need.

